# Bipolar disorders diagnosed in the elderly: Clinical and therapeutic particularities.

**DOI:** 10.1192/j.eurpsy.2023.1987

**Published:** 2023-07-19

**Authors:** M. Karoui, I. Kammoun, R. Kammoun, H. Nefzi

**Affiliations:** 1 Psychiatrie, Faculté de médecine de Tunis, Tunis, Tunisia

## Abstract

**Introduction:**

Data on the differences between young and elderly patients with bipolar disorder and between elderly patients with early and late age of onset are limited.

**Objectives:**

to study the clinical differencies between bipolar patients with an onset in old age and other bipolar patients.

**Methods:**

This is a retrospective study of 420 bipolar patients. In this study, patients with onset after age 60 (n=37) were compared to other patients with early-onset bipolar (EOB) (<>50 years; n=383).

**Results:**

In the year before recruitment, older patients more frequently reported a rapid course of the illness, but fewer suicide attempts , more often a single psychotropic medication and had less severe manic and psychotic symptoms, atypical antipsychotics were administered less frequently. but no difference in depressive symptomatology was observed.

**Conclusions:**

Elderly bipolar patients differ from younger bipolar patients in course and treatment. Medication use and the occurrence of rapid cycling in elderly bipolar patients deserve careful investigation.

**Disclosure of Interest:**

None Declared

